# Monthly Summary

**Published:** 1870-05

**Authors:** 


### MONTHLY SUMMARY.

*Hydrate of Chloral.*—Prof. W. A. Hammond, in a very interesting communication, says the first effect of hydrate of chloral is to cause congestion of the cerebral blood-vessels, and subsequently induce the very opposite condition. Small doses produce the first, large doses the latter state. A strong man with acute mania, quiet and not very irrational at the time got five grains hypodermically. He very soon became furiously excited, broke away from his attendants, rushed into the street, ran nearly a mile, and remained highly maniacal for several hours. The next day he had twenty grains hypodermically. He fell asleep in forty minutes after, and slept for ten hours. He had not previously slept for eleven days. He awoke free from delusions, and altogether better than he had been for several weeks. Forty grains of hydrate of chloral were administered to a gentleman suffering from cerebral hyperæmia and wakefulness, who had slept none the night before, and only about two hours a night for several weeks. In half an hour he was asleep and slept soundly for nearly twelve hours. Other cases almost similar in character and results are given.—*New York Medical Journal.*

---

*Death from Chloroform. Fatal Tooth Pulling.*—Mrs. Bradford Foote of Sheffield died from the effects of chloroform in the office of Dr. W. W. Rice, in Great Barrington, Mass., Saturday afternoon. Mrs. Foote called at the office to have a number of teeth extracted, and requested Dr. Rice to administer chloroform. He reluctantly consented to do so, as Mrs.Foote did not appear in perfect health. The chloroform was given by Dr. W. H. Parks, who was called in for that purpose. Twelve teeth were extracted, and Mrs. Foote had partly recovered consciousness, when she suddenly seemed to choke, aud died in a few moments.—*Boston Herald,*

*Almost Complete Ossification of the Human Body.* By WM. M. BYERS, M. D., Columbus, Colorado county, Texas.

B., a youth of 17, came under my notice first about four years ago with extensive ossification of the muscular system, which continued to progress till his death, which occurred a short time since. The early history of his case, as given by his family, is as follows:

He was a healthy, stout child at the age of eight months, at which time his parents removed to Texas by land. During their continuance on the road and camping out, they first discovered tumors about in different parts of his body, the size of small marbles, and very hard. These tumors gave apparently but little pain, and in no way affected the general health. After an interval of twelve or eighteen months the tumors began to disappear by absorption, and about the same time stiffness of the joints was manifested. Gradual anchylosis followed, and at the age of ten years the hip joints with those of the arms were completely anchylosed. The ossific matter began then to be deposited in the muscular system. The chest became enclosed with a complete sheet of bone, leaving no traces of outline of the ribs. The head was immovable fixed by the ossification of the sterno-cleido mastoideus on each side. The muscles of mastication were as yet unaffected, and his ability to chew his food not impaired. His digestion was good, and his mental faculties more than ordinary. He could as yet use wrists and hands, his armes being stiffened at right angles, thus allowing him to whittle a little with a pen-knife. When placed upon his feet he could move slowly and cautiously over a smooth surface, but if he got a fall, was unable to arise unassisted. Movement, however, as the anchylosis progressed, became more and more difficult and totally impossible at a latter period. He did not increase in stature after his tenth year, and never manifested any signs of virility. The forms of the muscles were perfectly preserved and could be distinctly felt through the skin, which adhered closely to them. The deposition of ossific matter continued during his life, and, when he died, he was almost complete bone. Up to the hour of his death his mind continued clear and unimpaired, and his digestion, until a short time previous, and was cheerful and apparently happy.—*New Orleans Journal of Medicine.*

*New Operations on the Lower Jaw—Lithotrity.*—To-day (March 9) we saw Mr Maunder, London Hospital, remove one-half the extent of the lower jaw through the mouth, without cutting the skin at all, and preserving a good share of the original periosteum. A myeloid tumour occupied the interior of the bone in the person of a girl 10 years of age. The bone was severed opposite the right canine tooth, and through the middle of the left ascending ramus.

The value of lithotrity was shown in the case of a man who, in eight sittings, had been relieved of two calculi; one had measured one and a half inch, the other one and three-eights of an inch.—*London Med. Times.*

---

*How to Cure a Cold.*—The following is extracted from a lecture by Dr. G. Johnson, the Professor of Medicine in King's College, and may prove interesting to our readers:

The popular domestic treatment consists in the use of a hot foot-bath at bed-time, a fire in the bedroom, a warm bed, and some hot drink taken after getting into bed, the diaphoretic action being assisted by an extra amount of bed clothes. Complete immersion in a warm bath is more efficacious than a foot-bath; but the free action of the skin is much more certainly obtained by the influence of hot air—most surely and profusely, perhaps, by the Turkish bath. The Turkish bath, however, is not always to be had, and even when available, its use in the treatment of catarrh is attended with some inconvenience. In particular, there is the risk of a too speedy check to the perspiration after the patient leaves the bath. On the whole, the plan which combines in the greatest degree of efficiency with universal applicability, consists in the use of a simple hot-air bath, which the patient can have in his own bedroom. All that is required is a spirit-lamp, with a sufficiently large wick. Such lamps are made of tin, and sold by most surgical instrument makers.

The lamp should hold sufficient spirit to burn for half an hour. The patient sits undressed in a chair with the lamp between his feet, rather than under the chair. An attendant then takes two or three blankets and folds them around the patient from his neck to the floor, so as to enclose him and the lamp, the hot air from which passes freely around the body. In from a quarter to half an hour there is usually a free perspiration, which may be kept up for a time by getting into bed between hot blankets. I have myself gone into a hot-air bath suffering from headache, pain in the limbs, and other indications of a severe incipient catarrh, and in the course of half an hour I have been entirely and permanently freed from these symptoms, by the action of the bath.

Another simple and sufficient mode of exciting the action of the skin consists in wrapping the undressed patient in a sheet wrung out of warm water, then, over this, folding two or three blankets. The patient may remain thus "packed" for an hour or two, until free perspiration has been excited.—*British Medical Journal.*

---

*A Snake in the Stomach.*—As an illustration of the absurd superstitions which obtain currency in the secular press, we produce the following "snake story" from the Evansville *Courier*:—

"A good deal of excitement was created in this city, yesterday, by the report that there was a negro woman in a house on Locust street, between Second and Third, who had a snake in her stomach; and a great many people visited the house. The negro woman came to Evansville about six days ago from Owensboro, Ky., and she states that she has suffered from the snake for about six years. At the pit of the stomach there is a large belt, almost the size of a man's arm; and just where the bones of the breast are separate, an impression is seen through the skin that resembles a snake's head. The belt, which extends around the body, if touched, produces the most excruciating pain to the woman, and as tears fall from her eyes, she cries, "don't make it bite me." On the touch, this belt, or snake, or whatever it may be, contracts, and then the woman suffers, it would appear, the pain of death. One thing singular is this: if the woman has plenty of milk, she experiences no particular pain, but rests quietly."

It is impossible to form a diagnosis from two symptoms exaggerated on hearsay evidence; but, allowing for magnified statements, such a story might arise from dilatation of the stomach with pyloric thickening and chronic ulcer.—*Medical Gazette.*

*Death Following the Application of Creasote to a Carious Tooth.* —*L'Imparziale* relates that a man aged 36, has lately died in the San Maria Nuova Hospital at Florence, from the results of the application of creasote to a carious tooth. Gingivitis and gangrene of the mouth appeared, and death from septicæmia took place in sixteen days. The relator of the fact mentions that when young, the free application of creasote to a carious tooth which he had, was followed by inflammation of the fauces and fever, by which he was confined to his bed for three days. These local effects ascribed to creasote are remarkable. We are not aware that any similar cases have been described as occurring in this country.—*Brit. Med. Jour.*

---

*Squills as a Poison for Rats.*—According to the French "Moniteur" there are in France upwards of two thousand millions of rats and other rodents. Supposing each of these little quadrupeds to commit the damage of only one centime per annum, this loss would amount in the aggregate to twenty millions of francs annually. Hence it is most desirable to find some means of destroying this vermin in large numbers as expeditiously as possible. Nux vomica, arsenic, phosphorus, and traps have been successively tried, but with no very decided success, and certainly not equal to the rate of increase of these prolific creatures. Recent experiments, however, show that squills (*Scilla Maritima,*) the bulb of which is much used in medicine, is not only a powerful poison for rodents, but also one they are very fond of. The way of preparing it for the desired purpose is as follows:—One of the bulbs is cut into slices, hashed and bruised, then done in the pan with fat, which is afterwards strained through a cloth and poured into broken plates and saucers to be placed in the cellars and other places infested with rats, mice, &c. To prevent dogs and poultry from eating of this poisonous compound in stables, pigeon-houses, or farm-yards, it may be put into a wooden box, about a foot and a half long, and having a hole at each end. The rat gets in at one end and goes out at the other, after partaking of the noxious food, which soon kills it. Squills may also be reduced to powder for the same purpose by bruising them in a mortar to a pulp, which is afterwards incorporated with as much flour as it will hold. This paste is then rolled out, as they do for a pudding, then cut into shreds, which are left to dry on hurdles or on sheets of pasteboard, and are afterwards pounded in a mortar. The powder thus obtained will keep for years, and may be put into boxes or barrels. If manufactured on a large scale, it may become a profitable article of exportation. In Algeria squills cost nothing, the country being absolutely overrun with them.—*Lond. Phar. Journ.*

---

*Influence of Sewing-Machines on Health.*—Some prejudice has been excited against the use of sewing-machines on the score of their injurious effects on health. As the result of some investigation on the subject, we may avow our conviction that these statements have been greatly exaggerated. They apply, we believe, chiefly to those worked by one foot only; and we are quite unable to discover that there is any kind of objection on the score of health to those in which both feet are employed. In America, where the use of machines is yet more common than with us, the attention of medical men has been a good deal directed to the subject, and with, we believe, tolerable unanimity of result. Against the double pedal-machine in common use in England, as far as we can learn from inquiry amongst those who have had them for many years in constant employment, there is not the slightest evidence. Of their sanitary advantages in saving time from a sedentary and very tiresome occupation, and thus favoring relaxation and exercise, it is not nocessary that we should speak.—*Brit. Med. Jour.*

---

*Bichloride of Methylene.*—Dr. Charles Bell Taylor, in the course of a paper on cataract extraction (*British Medical Journal*) calls attention to the rapidity with which anaesthesia is induced by the bichloride of methylene. When given with an inhaler, from which air is almost entirely excluded, insensibility is caused in half a minute, and the effect passes off in two or three minutes. It is said to be quite equal in its properties to nitrous oxide for slight operations, such as tooth-extraction, and, indeed, has long been used by a dentist in Brighton.—*Med. Gazette.*

*Doses for Hypodermic Use.*—In answer to the many inquiries made by correspondents concerning the medicines and their doses, to be used by hypodermic injection, we insert a table prepared by a young physician of this city for a new Visiting-list, just issued here by another physician, also of this place :

ACONITE (*Aconitia, Off.*—R. Aconitiæ, gr. j ; aquæ destillatæ purissimæ, f. ʒ ss. Misce et fiat solutio. DOSE—*minimum,* M· ij=$\frac{1}{120}$ gr. : *maximum,* M. iv=$\frac{1}{60}$ gr.

ATROPINE (*Atropia, Off.*)—R. Atropiæ sulphatis, gr. j ; aquæ destillatæ pur.; f. ʒ ss. Misce et fiat solutio. DOSE—*minimum,* M. ij=$\frac{1}{120}$ gr. : *maximum,* M: iv=$\frac{1}{48}$ gr, Best of all medicinal remedies for every kind of pain in pelvic viscera.

CAFFEINE, THEINE.—℞. Caffeinæ, gr. ij ; aquæ destilatæ bullientis, M. lxxx. Misce et fiat solutio. DOSE—*minimum,* M. 20=$\frac{1}{2}$ gr. : *maximum,* M. 40=1 gr. Neuralgia and alcoholic insomnia.

STRYCHNINE (*Strychnia, Off.*)—℞. Strichniæ sulphatis, gr. ij ; aquæ destillatæ bullientis, ℥ j. Misce et fiat solutio. DOSE—*minimum,* M. ij=$\frac{1}{120}$ gr. : *maximum,* M. iv=$\frac{1}{60}$ gr. Gastralgia and neuralgia of heart.

MORPHINE (*Morphia, Off.*)—℞. Morphiæ acetatis, gr. v ; acidi acetici, M. j ; aquæ destillatæ bullientis, f. ʒ j. Misce et fiat solutio. DOSE—*minimum,* M. j=$\frac{1}{12}$ gr. : *maximum,* M. vj=$\frac{1}{2}$ gr. Ten grains to a drachm can be dissolved by use of glycerine. Especially useful in delirium tremens and neuralgic pains.

NICOTINE (*Nicotina*—℞. Nicotinæ, gr. j ; aquæ destillatæ puriss., f. ℥ ss. Misce et fiat solutio. DOSE—*minimum,* M. ij=$\frac{1}{120}$ gr. : *maximum,* M. iv=$\frac{1}{60}$ gr. Useful in tetanus.

DIGITALINE (*Digitalia, Off.*)—℞. Digitaliæ, gr. j ; aquæ desillatæ purissimæ, f. ℥ ss. Misce et fiat solutio. DOSE—*minimum,* M. ij=$\frac{1}{120}$ gr. : *maximum,* M. iv=$\frac{1}{60}$ gr. Useful in febrile conditions.

CALABAR BEAN (*Physostigma Venenosum.*)—℞- Extractum physostigmatos venenosi alcoholici, gr. ij ; aquæ distillatæ purissimæ, ʒ ij ; glycerinæ, ℥ j. Misce et fiat solutio. DOSE—*minimum,* M. 20=$\frac{1}{15}$ gr. : *maximum,* M. 40=$\frac{1}{8}$ gr. In tetanus—*American Practitioner.*—*Am. Electic Med. Review.*

